# Experimental estimation of energy absorption during heel strike in human barefoot walking

**DOI:** 10.1371/journal.pone.0197428

**Published:** 2018-06-28

**Authors:** Patricia M. Baines, A. L. Schwab, A. J. van Soest

**Affiliations:** 1 Department of Mechanical Engineering, Delft University of Technology, Delft, Netherlands; 2 Department of Human Movement Sciences, Vrije Universiteit Amsterdam, Amsterdam, Netherlands; 3 Research Institute Amsterdam Movement Sciences, Amsterdam, Netherlands; Universite de Nantes, FRANCE

## Abstract

Metabolic energy expenditure during human gait is poorly understood. Mechanical energy loss during heel strike contributes to this energy expenditure. Previous work has estimated the energy absorption during heel strike as 0.8 J using an effective foot mass model. The aim of our study is to investigate the possibility of determining the energy absorption by more directly estimating the work done by the ground reaction force, the force-integral method. Concurrently another aim is to compare this method of direct determination of work to the method of an effective foot mass model. Participants of our experimental study were asked to walk barefoot at preferred speed. Ground reaction force and lower leg kinematics were collected at high sampling frequency (3000 Hz; 1295 Hz), with tight synchronization. The work done by the ground reaction force is 3.8 J, estimated by integrating this force over the foot-ankle deformation. The effective mass model is improved by dropping the assumption that foot-ankle deformation is maximal at the instant of the impact force peak. On theoretical grounds it is clear that in the presence of substantial damping that peak force and peak deformation do not occur simultaneously. The energy absorption results, due the vertical force only, corresponding to the force-integral method is similar to the results of the improved application of the effective mass model (2.7 J; 2.5 J). However the total work done by the ground reaction force calculated by the force-integral method is significantly higher than that of the vertical component alone. We conclude that direct estimation of the work done by the ground reaction force is possible and preferable over the use of the effective foot mass model. Assuming that energy absorbed is lost, the mechanical energy loss of heel strike is around 3.8 J for preferred walking speeds (≈ 1.3 ^m^/_s_), which contributes to about 15–20% of the overall metabolic cost of transport.

## Introduction

The metabolic cost of human walking is substantial. Not only has it been reported to account for about 25% of the daytime energy expenditure of an office clerk [1], it is also well established that walking distance of many people with a locomotor impairment, for instance following a stroke, is limited due to the associated metabolic cost [2–4]. Thus, it is important for the evaluation and treatment of gait disorders to have a thorough understanding of the processes underlying the metabolic energy expenditure during human locomotion, and to be able to measure the energy associated with these processes [5]. It is accepted that positive and (to a lesser extent) negative muscle fiber mechanical work contribute to the metabolic cost of walking [6]. While it is debated in which phase positive muscle fiber mechanical work is primarily done [6–12], it is accepted that, during steady-motion level walking, the positive muscle fiber mechanical work serves to compensate for negative muscle fiber mechanical work, for negative work associated with air friction (generally assumed to be negligible), and for mechanical energy lost in the foot-ground contact. There has been recent literature that indicates that soft tissue deformation could play a larger role in cost of transport that was previously thought [13, 14]. For running, [14] estimate about 19 J per stance phase during running at 3 ^m^/_s_ associated with soft tissue energy loss. [13] estimated a total 13 J of energy expenditure in the collision phase of walking, where around 7 J could not be accounted for by modeled joint or segment work and thought to be attributed by soft tissue deformation. We want to build on these findings and further investigate a major contributor to this unaccounted energy loss during the collision phase of walking.

In this study, we focus on quantification of the mechanical energy absorbed in the foot-ground contact during steady motion horizontal walking, and in particular on the energy exchange between heel and ground that occurs during heel strike, i.e. during the first 10–50 ms of the double support phase. [15, 16]. We define the heel strike to start at heel contact and to end when the vertical foot deformation is maximal or equivalently when the vertical foot deformation speed is zero. Where others, primarily motivated by experimental limitations, defined heel strike to end at 90% or 100% of the impact transient peak seen in the vertical ground reaction force [15, 16]. [15] argued that direct experimental estimation of the energy absorbed during heel strike is difficult because it is hard to achieve the required tight synchronization between data on ground reaction force (GRF) and kinematics. As an alternative, these authors analyzed an effective foot mass model similar to the one proposed by [17], and suggested that during heel strike, the behavior is governed by the effective foot mass *M*_eff_ that is coupled visco-elastically to the ground. Assuming that the velocity of the effective foot mass is zero at the instant of the impact peak in the vertical GRF, they estimated the mechanical energy absorption during heel strike to be 0.8 J. Assuming a muscle mechanical efficiency of 20% for positive muscle fiber mechanical work [18] and assuming all of the energy absorbed by the heel pad is lost, this value would explain about 4% of the metabolic cost per step during steady-motion level walking (defined according to [6]). This suggests that energy loss during heel strike is negligible.

We will show that direct experimental estimation of the mechanical energy absorption during heel strike is possible, using a force-integral method. We will compare the results of the force-integral method to the results obtained using both the original and an improved application of the effective mass model.

## Methods

Participants were asked to walk barefoot at their preferred walking speed on level ground in a large hall where a force platform was mounted in the floor. Ten trials were recorded for each participant. The experiment was focused on heel contact of the right foot. GRF and lower leg kinematics were collected at high sampling frequency, with tight synchronization. This data was used to estimate the work done by the GRF and the energy absorption corresponding to the original and an improved application of the effective mass model.

All of the raw data, the processed data, a high speed video made during the pilot experiments together with Matlab scripts used for processing and generating figures can be found in our online repository [19].

### Participants

In total 12 (4 male, 8 female) healthy participants (age: 27 ± 5 years; mass: 76.7 ± 15 kg; BMI: 24.5 ± 5.0 ^kg^/_m^2^_) were involved in the experiment. Prior to the experiment, the participants were asked to sign an informed consent form. The experiment was approved by the ethical board of the Human Movements Sciences Faculty at the Free University of Amsterdam.

### Instrumentation

**Fig 1:**
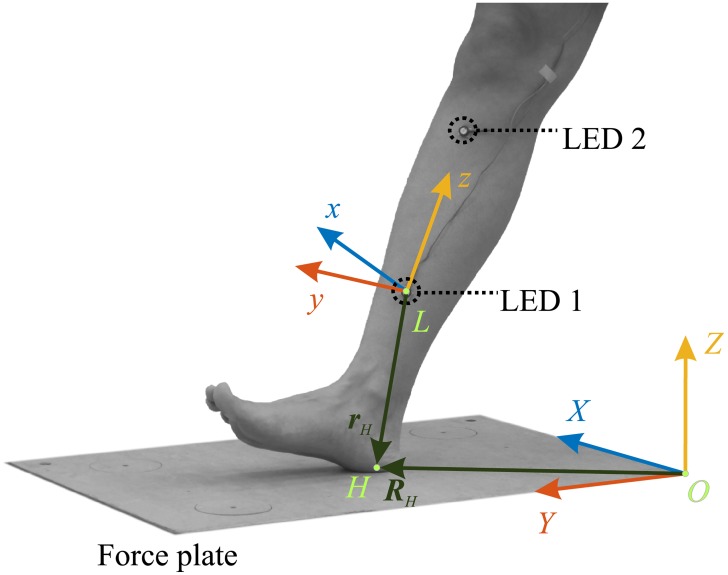
Schematic of the lower leg and force plate, showing coordinate systems and heel point. A global coordinate system *XYZ* is defined, where the origin *O* is situated in a corner of the force plate. The direction *X* is lateral, *Y* is anterior and *Z* is vertical up. A local coordinate system *xyz* is defined, where the origin *L* is situated at an LED kinematic marker. This is the lower of two markers placed directly on the skin over the medial surface of the tibia. The local *z* direction points to the second LED marker, the y direction is perpendicular to the *z* axis and lies in the plane of progression (defined as a plane parallel to the global *YZ*-plane), the *x* direction is defined orthogonal to the *yz*-plane to complete the local right-handed orthogonal coordinate system. The position of the heel point *H* is derived from the location of the center of pressure as obtained from the force plate data during heel strike, as measured in the global coordinate system *XYZ*. These global center of pressure points are transformed to the local coordinate system *xyz*. The median *xyz*-position of these transformed local center of pressure points is taken as the position of the heel point ***r***_*H*_, which is a time-invariant vector in the local lower-leg coordinate system *xyz*. The global position of the heel point ***R***_*H*_ is time variant, due to the movement of the local coordinate system in the global coordinate system.

GRF was measured using a piezoelectric Kistler force plate (Type 9282B11) mounted permanently in the floor, with a sampling frequency of 3000 Hz. Deformation due to heel strike is primarily attributed to the soft tissue in the heel pad, however [20] has shown by comparing in vivo and in vitro tests of the heel pad and lower leg that deformation of the rest of the lower leg cannot simply be neglected in lower extremity impact tests. Therefore we chose to take the entire foot-ankle deformation into account. The kinematics of 2 LED’s were measured using an Optotrak Certus motion capture system, with a sampling frequency of 1295 Hz. The number of Optotrak LEDs was reduced to two in order to maximize Optotrak sample frequency. As the ankle joint angle has been reported to remain more or less constant during heel strike [15], the two LEDs were placed on the skin over the medial surface of the tibia, with an inter-LED distance of around 0.16 m (see Fig 1); skin movement artifact at this location has been reported to be very small [21]. The movement of these LEDs was used to describe the foot-ankle deformation, which is defined as the deformation between the heel contact point and the tibia.

During pilot experiments, an impact test was used to determine the error in synchronization between force measurements and measurements of kinematics. The maximum synchronization error was found to be less than 0.001 s. During pilot experiments the lower leg motion during heel strike was also captured using a high speed camera (sampling frequency of 1200 Hz) in order to determine if the constant ankle angle assumption during heel strike holds (for video see [19]). Visual inspection of the images during heel strike supported the assumption that the change in ankle angle during heel strike is negligible.

### Data analysis

Optotrak data was filtered using a low pass forward-backward filter (cut-off frequency 100 Hz) in order to remove high frequency noise. Two coordinate systems were used, the first being the global *XYZ* coordinate system with origin *O*, the second being the local *xyz* coordinate system with origin *L* that moves with the rigid part of the foot-lower-leg system (see Fig 1). This local system was used to define a heel point *H*, the position of which is constant in this local *xyz* coordinate system (***r***_*H*_ is constant). The net GRF, as well as the *X* and *Y*-coordinate of the point of application of the vertical component of the GRF (’center of pressure’ COP) were calculated using standard methods using the 8 Kistler force plate output channels (4 for the vertical force components and 2 for each horizontal component corresponding to the lines of action through the four sensors embedded in the force plate) [22, 23].

The location of the heel point *H* was defined using this COP position relative to the global *XYZ* coordinate system as a basis. First the time-variant COP position was transformed from the global *XYZ* coordinate system to the local *xyz* coordinate system by establishing and subsequently applying the time-variant rotation and translation between these coordinate systems [24]. The median *xyz*-position of these local COP positions surrounding heel impact was taken as the constant location of the heel contact point ***r***_*H*_ relative to the local *xyz* coordinate system. The first sample included in this calculation was the first sample in which the vertical GRF exceeded 25% of the impact peak force; the last sample included in this calculation was chosen such that the impact peak is in the middle of the samples used. The ***r***_*H*_ vector was calculated for each trial separately. The time-variant location of the heel point ***R***_*H*_ relative to the global *XYZ* coordinate system is found by applying the inverse time-variant rotation and translation readily established between the global and local coordinate system to the constant ***r***_*H*_ [24].

#### Force-integral method

**Fig 2:**
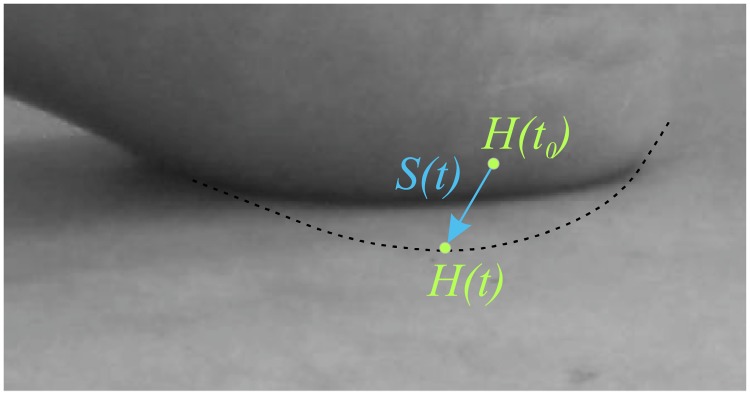
Schematic zoomed in on heel-pad-ground contact, showing foot-ankle deformation definition. The global position of the heel point *H*, defined with position vector ***R***_*H*_ with respect to the global coordinate system *XYZ* as shown in Fig 1, is shown at the instant of initial heel contact *t*_0_ and at a time *t* during heel strike. Due to deformation of the heel pad, foot and ankle, the heel point *H*(*t*) will effectively move through the ground during heel strike. The movement of the heel point during heel strike is taken as a measure for the foot-ankle deformation ***S***(*t*)(= ***R***_*H*_(*t*) − ***R***_*H*_(*t*_0_)).

Direct estimation of the work done by the GRF during heel strike requires that foot-ankle deformation was determined from the kinematic data. To that end, we have defined a ‘heel point’ *H*, the position of which is time invariant relative to the *xyz* coordinate system (***r***_*H*_) and time variant relative to the *XYZ* coordinate system (***R***_*H*_). We assumed that foot-ankle deformation ***S*** equals the displacement of this heel point *H* relative to the *XYZ* coordinate system (***S***(*t*) = ***R***_*H*_(*t*) − ***R***_*H*_(*t*_0_), where *t*_0_ is the instant of heel strike at which GRF is zero) (see Figs 1 and 2). Subsequently, the displacement of this heel point relative to the *XYZ* coordinate system was calculated from the kinematic data. Finally, energy *W* absorbed by the foot-ankle using the force-integral method was calculated by numerically integrating the GRF vector ***F*** over the foot-ankle deformation vector ***S***:

$$\begin{array}{c} W=\int_{S(t_{0})}^{S(t_{e})}F\cdot dS \end{array}$$ (1)

$$\begin{array}{c} =W_{X}+W_{Y}+W_{Z} \end{array}$$ (2)

$$\begin{array}{c} =\int_{S_{X}\left(t_{0}\right)}^{S_{X}\left(t_{e}\right)}F_{X}\;dS_{X}+\int_{S_{Y}\left(t_{0}\right)}^{S_{Y}\left(t_{e}\right)}F_{Y}\;dS_{Y}+\int_{S_{Z}\left(t_{0}\right)}^{S_{Z}\left(t_{e}\right)}F_{Z}\;dS_{Z} \end{array}$$ (3)

Where *t*_*e*_ is the instant when the vertical heel velocity is zero (*Ż*_*H*_(*t*_*e*_) = 0). We refer to the interval from *t*_0_ to *t*_*e*_ as ‘heel strike’. The values of these integrals were determined using a trapezoidal method.

#### Improved application of the effective mass model

The energy change was also determined using the effective foot mass *M*_eff_ model [15, 17]. The *M*_eff_ in the effective foot mass model represents the part of the body mass that participates in the initial phase after foot-ground impact and is only acted upon by the GRF. The effective foot mass model is concerned only with motions and forces in the vertical *Z* direction. In the original effective mass model, it is also assumed that the period of heel strike ends at the time of the ‘impact peak’ (*t*_*p*_) in the GRF and it is further assumed that the vertical velocity *Ż*_*H*_ is zero at the impact peak time *t*_*p*_, making the model applicable to kinematic and force data that are not synchronized [15]. Below we will argue that, due to the viscous properties of the heel pad, the impact peak in the force data does not occur at zero vertical heel velocity. Since we have kinematic and force data which is tightly synchronized, we are not forced to make this assumption. Therefore in this improved application of the *M*_eff_ model, we used the synchronized kinematic data to identify the end of heel strike to be at *Ż*_*H*_(*t*_*e*_) = 0. The effective foot mass corresponding to this improved application of the model *M*_*e*_ was determined from the impulse-momentum equation:

$$\begin{array}{c} \int_{t_{0}}^{t}F_{Z}\;dt=M_{\text{eff}}\;\left({Ż}_{H}\left(t\right)-{Ż}_{H}\left(t_{0}\right)\right)+M_{\text{eff}}\;g\;\left(t-t_{0}\right) \end{array}$$ (4)

$$\begin{array}{ccc} \to M_{\text{eff}} & = & \frac{\int_{t_{0}}^{t}F_{Z}\;dt}{{Ż}_{H}\left(t\right)-{Ż}_{H}\left(t_{0}\right)+g\;\left(t-t_{0}\right)}\\ \text{evaluated}\;\text{until}\;t_{e} & & \end{array}$$ (5)

$$\begin{array}{ccc} \to M_{e} & = & \frac{\int_{t_{0}}^{t_{e}}F_{Z}\;dt}{-{Ż}_{H}\left(t_{0}\right)+g\;\left(t_{e}-t_{0}\right)} \end{array}$$ (6)

Where *Ż*_*H*_(*t*_0_) and *Ż*_*H*_(*t*_*e*_) are the vertical heel velocities at the beginning and end of heel strike respectively, which are ascertained by using a gradient approach on the vertical heel point position *Z*_*H*_ (which is the vertical component of ***R***_*H*_), and *g* is the gravitational acceleration (taken as *g* = 9.81 ^m^/_s^2^_). The total change in energy Δ*E*_*e*_, consisting of a change in kinetic Δ*E*_kin_ and potential Δ*E*_pot_ energy, of this *M*_*e*_ were determined in order to indirectly determine work *W* done on the heel pad.

$$\begin{array}{c} W=\Delta E=\Delta E_{\text{kin}}+\Delta E_{\text{pot}} \end{array}$$ (7)

$$\begin{array}{ccc} & = & \frac{1}{2}\;M_{\text{eff}}\;\left({Ż}_{H}{\left(t_{0}\right)}^{2}-{Ż}_{H}{\left(t\right)}^{2}\right)+M_{\text{eff}}\;g\;\left(Z_{H}\left(t\right)-Z_{H}\left(t_{0}\right)\right)\\ \text{evaluated}\;\text{until}\;t_{e} & & \end{array}$$ (8)

$$\begin{array}{c} \to \Delta E_{e}=\frac{1}{2}\;M_{e}\;{\left({Ż}_{H}\left(t_{0}\right)\right)}^{2}+M_{e}\;g\;\left(Z_{H}\left(t_{e}\right)-Z_{H}\left(t_{0}\right)\right) \end{array}$$ (9)

Where *t*_*e*_ is end time of heel strike and (*Z*_*H*_(*t*_*e*_) − *Z*_*H*_(*t*_0_)) is the maximal vertical foot-ankle deformation, which is the vertical component of ***S***(*t*_*e*_).

In order to investigate the influence of this improvement on the application of the effective mass model and be able to compare our results to [15], we evaluated the original model under the assumption that heel strike ends at the instant of the impact force peak *t*_*p*_ and that *Ż*_*H*_(*t*_*p*_) = 0 to estimate the corresponding effective mass *M*_*p*_*CS*_ and energy absorption due to heel strike Δ*E*_*p*_*CS*_ and also evaluated the improved application of the effective mass model until the instant of the impact force peak *t*_*p*_ (where *Ż*_*H*_(*t*_*p*_) ≠ 0) to estimate the corresponding effective mass *M*_*p*_ and energy absorption due to heel strike Δ*E*_*p*_.

### Statistical analysis

The results of the 10 trials per participant were grouped together and the mean and standard deviation were calculated of these groups. The mean of the subject means gives the overall mean, the mean of the subject standard deviations was used as a measure for the intrasubject variability and the standard deviation of the subject means was used as a measure for the intersubject variability.

A Kolmogorov-Smirnov test on the subject means for the energy absorption during heel strike (*W*, *W*_*Z*_, Δ*E*_*e*_, Δ*E*_*p*_ and Δ*E*_*p*_*CS*_) indicated that the underlying distributions are unlikely to be normal (p(*W*) = 2.8E-10, p(*W*_*Z*_) = 1.9E-9, p(Δ*E*_*e*_) = 2.1E-9, p(Δ*E*_*p*_) = 7.6E-7, p(Δ*E*_*p*_*CS*_) = 1.8E-5). Therefore a Friedman test was performed to test the statistical significance of the difference between the methods. Wilcoxon signed rank tests were used to investigate specific differences. For these paired tests a Bonferroni correction was applied to set the level of significance to 0.01.

### Parameter sensitivity analysis

A parameter sensitivity analysis was performed on the estimation ${\hat{r}}_{H}$ of the heel point *H* position ***r***_*H*_ in the local coordinate system. A new heel point *Q* was defined, which is located approximately 1.7 cm away from point *H* by moving 1 cm downwards (-*z*), 1 cm posterior (-*y*) and 1 cm lateral (*x*), which is assumed to be further away than any reasonable inaccuracies expected in ascertaining the heel point *H*. In order to verify the validity of this assumption, we have compared the location of this point *Q* to the various estimated locations of the COP points during heel strike in the local *xyz* coordinate system. Point *Q* was used in order to calculate the energy absorption during heel strike in the same manner as for point *H* and the results for these two points were compared.

## Results

During heel strike, the variation in inter-marker distance was in the same order of magnitude as the accuracy of the Optotrak system (0.1 mm). The preferred walking speed of the participants was consistent over trials (Table 1), and the average value was consistent with previous research [25].

**Table 1:** Energy absorption, foot-ankle deformation and other experimental results.

	overall mean	range of subject means	subject variability (intra; inter)
walking speed (in m/s)	1.3	(1.1; 1.5)	(0.065; 0.14)
step frequency (in Hz)	1.9	(1.7; 2.1)	(0.18; 0.17)
|***S***(*t*_*e*_)| (in mm)	21.2	(13.4; 32.0)	(3.0; 6.0)
*S*_*X*_(*t*_*e*_) (in mm)	−6.8	(−15.5; −0.51)	(1.8; 3.8)
*S*_*Y*_(*t*_*e*_) (in mm)	14.2	(6.4; 27.2)	(3.8; 6.9)
*S*_*Z*_(*t*_*e*_) (in mm)	−12.9	(−15.5; −9.5)	(0.77; 1.9)
*Ż*_*H*_(*t*_0_) (in m/s)	−0.57	(−0.78; −0.39)	(0.067; 0.12)
*Ż*_*H*_(*t*_p_) (in m/s)	−0.52	(−0.75; −0.35)	(0.044; 0.14)
(*Ż*_*H*_(*t*_p_) − *Ż*_*H*_(*t*_0_)) (in m/s)	0.045	(−0.068; 0.14)	(0.043; 0.058)
*t*_*p*_ (in ms)	17.5	(12.0; 23.3)	(1.8; 3.6)
*t*_*e*_ (in ms)	29.8	(20.0; 45.2)	(4.3; 7.6)
*t*_*e*_/*t*_*p*_ (−)	1.70	(1.47; 2.28)	(0.13; 0.21)
*W* (in J)	−3.8	(−6.9; −1.4)	(0.47; 1.7)
*W*_*X*_ (in J)	−0.75	(−2.2; 0.016)	(0.36; 0.65)
*W*_*Y*_ (in J)	−0.36	(−0.88; −0.016)	(0.22; 0.31)
*W*_*Z*_ (in J)	−2.7	(−4.6; −1.2)	(0.30; 1.1)
Δ*E*_*e*_ (in J)	−2.5	(−3.8; −1.2)	(0.31; 0.88)
Δ*E*_*p*_ (in J)	−1.4	(−2.3; −0.59)	(0.61; 0.13)
Δ*E*_*p*_*CS*_ (in J)	−0.84	(−1.5; −0.38)	(0.071; 0.36)
*M*_*e*_ (in body mass %)	11.1	(8.6; 19.5)	(1.9; 2.8)
*M*_*p*_ (in body mass %)	17.6	(9.3; 46.8)	(11.8; 4.6)
*M*_*p*_*CS*_ (in body mass %)	4.1	(3.5; 5.2)	(0.45; 0.56)

Estimation of the overall mean, range of subject means, intra- and intersubject variability of the forward walking speed, step frequency, the magnitude |***S***(*t*_*e*_)|, lateral *S*_*X*_(*t*_*e*_), anterior *S*_*Y*_(*t*_*e*_) and vertical *S*_*Z*_(*t*_*e*_) component of the foot-ankle deformation, vertical heel velocity at start of heel strike *Ż*_*H*_(*t*_*0*_) and instant of peak force *Ż*_*H*_(*t*_*p*_), time to peak force *t*_*p*_, heel strike duration *t*_*e*_, energy absorption per heel strike using the force-integral method *W*, the force-integral method for ground reaction force components (*F*_*X*_, *F*_*Y*_, *F*_*Z*_) *W*_*X*_, *W*_*Y*_, *W*_*Z*_, the improved application of the effective foot mass model Δ*E*_*e*_ and Δ*E*_*p*_, the original application of the effective foot mass model following Chi and Schmitt [15] Δ*E*_*p*_*CS*_ and corresponding effective foot mass calculated until end of heel strike *M*_*e*_, until peak force *M*_*p*_ and using original assumption *M*_*p*_*CS*_,.

**Fig 3:**
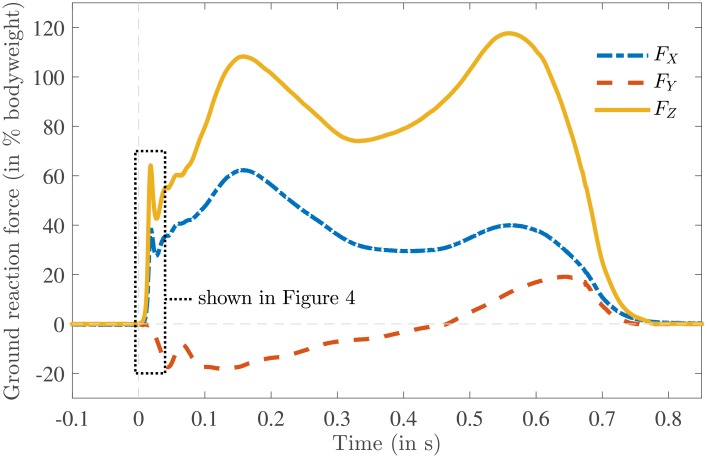
A typical example of the measured ground reaction force acting on the right foot defined in the global coordinate system *XYZ* as a percentage of body weight (727 N) as a function of time, where the subscript *X* corresponds to mediolateral, *Y* to anterior-posterior and *Z* to vertical component of the force. A dotted rectangle is shown to indicate the initial part of the stance phase where heel strike takes place. The ground reaction forces of this initial part is shown in Fig 4.

**Fig 4:**
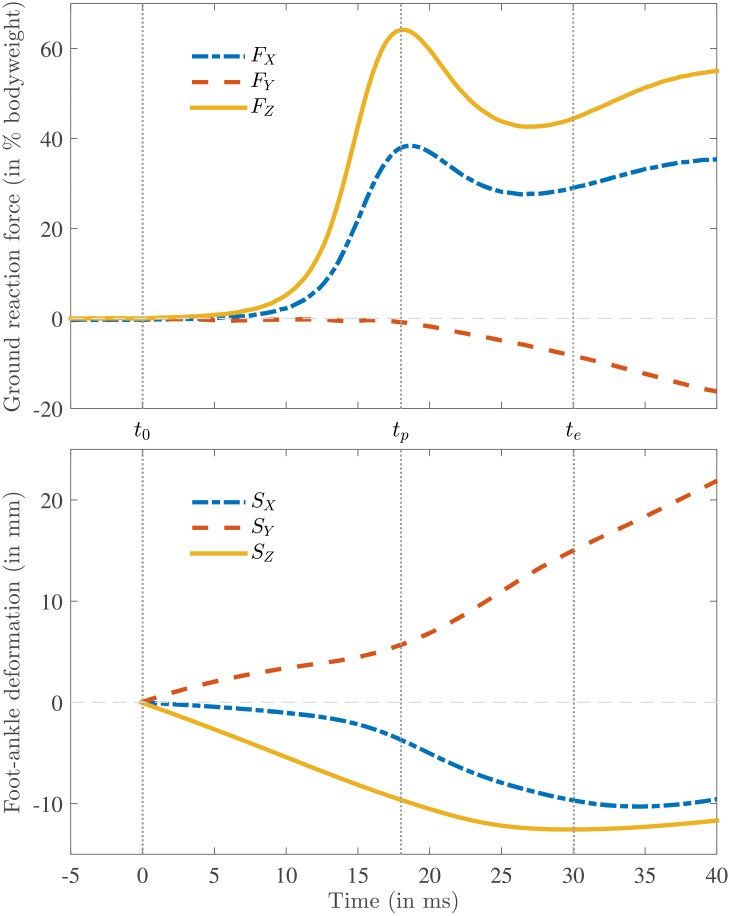
A typical example of the measured ground reaction force acting on the heel (top) and foot-ankle deformation (bottom) as a function of time defined in the global coordinate system *XYZ* (shown in Fig 1), where the subscript *X* corresponds to mediolateral, *Y* to anterior-posterior and *Z* to vertical component (same measurement as in Fig 3). The start of heel strike is shown by a vertical dotted line indicated by *t*_0_, the instant of the vertical force peak is indicated by *t*_*p*_ and the end of heel strike, which is defined as zero vertical heel velocity, by *t*_*e*_.

**Fig 5:**
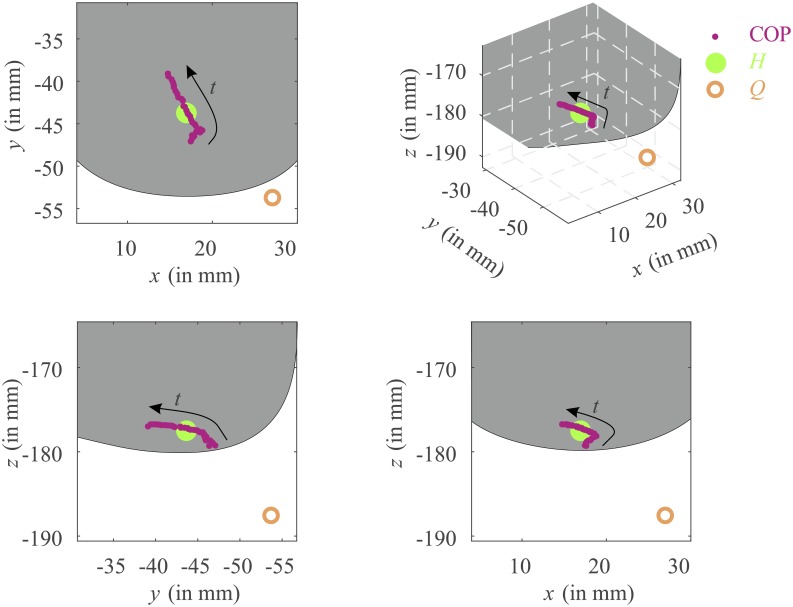
A typical example of the location in the local *xyz* coordinate system of the center of pressure (COP) points during the impact phase used to define the heel point *H*. The COP point at each instant is transformed to the local *xyz* coordinate system (see Fig 1), where the mean of these points are used to define the heel point *H*. The progression of the COP points as a function of time is indicated by the arrow. A point *Q* is defined in order to perform a parameter sensitivity analysis. This point lies 1 cm away from *H* in lateral (*x*), posterior (−*y*) and inferior (−*z*) direction. The gray areas are drawn to roughly indicate the boundaries of the undeformed heel.

A typical GRF measurement is shown in Fig 3. In this study we were interested only in the early part (≈ 0.03 s) of the double support phase (≈ 0.1 s), where the heel strike occurs. In Fig 4 the GRF is shown together with the foot-ankle deformation for the same measurement as Fig 3. The zero vertical heel velocity occurred substantially later than the impact peak; this substantial difference between *t*_*p*_ and *t*_*e*_ was seen in all the measurements (Table 1). As can be seen from Fig 4, the vertical foot-ankle deformation indicates that the vertical heel velocity remained more or less constant during the initial part of heel strike (Table 1: on average *Ż*_*H*_(*t*_0_) = −0.57 ^m^/_s_ and *Ż*_*H*_(*t*_*p*_) = −0.52 ^m^/_s_) and decreased quickly after the impact force peak. The maximum vertical foot-ankle deformation *S*_*Z*_(*t*_*e*_) was around −13 ± 2 mm, which is a compressive deformation. The corresponding shear deformations *S*_*X*_(*t*_*e*_) and *S*_*Y*_(*t*_*e*_) were −7 ± 4 mm and 14 ± 7 mm respectively, together bringing the total foot-ankle deformation at the end of heel strike to |***S***(*t*_*e*_)| = 21 ± 6 mm. Fig 5 shows the progression of the COP in the local *xyz* coordinate system together with the defined heel point *H* and point *Q* used for a parameter sensitivity analysis (see Section Parameter sensitivity analysis). This figure shows that the point *H* was located within the area of the COP samples and point *Q* was located at a considerable distance from it.

Using the force-integral method (Eq 1) the total work done by the GRF vector was found to be *W* = −3.8 ± 1.7 J. Herein the largest component was the vertical component *W*_*Z*_ = −2.7 ± 1.1 J, then the lateral component *W*_*X*_ = −0.8 ± 0.7 J and the anterior component *W*_*Y*_ = −0.4 ± 0.3 J. For some trials there were components of this calculated work which had a small positive value (6% for *W*_*X*_, 13% for *W*_*Y*_, 0% for *W*_*Z*_ of all trials), which results in a small positive mean value for a single subject for a single component (subject 3: *W*_*X*_ = 0.016 J), which must be due to measurement and/or modeling errors.

The original effective foot mass model predicted an energy absorption of Δ*E*_*p*_*CS*_ = −0.8 ± 0.4 J, with corresponding foot mass *M*_*p*_*CS*_ = 4.1 ± 0.6% of bodyweight. The improved application of the effective foot mass model resulted in a substantially higher energy absorption of Δ*E*_*p*_ = −1.4 ± 0.1 J, with corresponding foot mass *M*_*p*_ = 18 ± 5% of bodyweight and Δ*E*_*e*_ = −2.5 ± 0.9 J, with corresponding foot mass *M*_*e*_ = 11 ± 3% of bodyweight.

In this study the overall difference between the methods of determining energy absorption due to heel strike (*W*, *W*_*Z*_, Δ*E*_*e*_, Δ*E*_*p*_, Δ*E*_*p*_*CS*_) was found to be statistically significant (*χ*^2^(59) = 45.9, p = 2.6E-9). Where the result of the vertical component of the force-integral method (*W*_*Z*_) and the improved application of the effective foot mass model (Δ*E*_*e*_) did not differ significantly (Z = −1.2, p = 0.24), however all the other method combinations did show statistically significant differences (Z = −3, p = 2.2E-3).

In order to determine the sensitivity of the results for the choice of heel point, results were also calculated for point Q (see Section Parameter sensitivity analysis); energy absorption for point Q was found to be 2–5% smaller than for the default heel point and results of statistical tests were similar.

**Fig 6:**
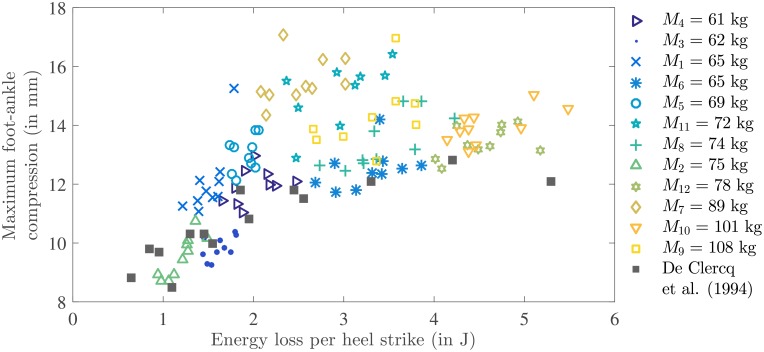
Subject specific scatter plot of maximal compressive foot-ankle deformation |*S*_*Z*_*e*__| as a function of corresponding energy absorption due to heel strike *W*_*Z*_, which is estimated using force-integral method (integral of vertical ground reaction force over vertical foot-ankle deformation). Each marker represents a single measurement, where the marker style is participant specific and the body mass is shown in the legend for comparison. Previous results from in vivo tests on the human barefoot constrained lower legs collected by [26] are shown.

In Fig 6 the relation between maximum vertical foot-ankle compression |*S*_*Z*_(*t*_*e*_)| and energy absorption per heel strike *W*_*Z*_ is shown together with experimental results collected by [26] (from [27–30]). In these studies, in vivo tests were performed on human constrained lower legs with bare feet to determine compressive mechanical properties of the heel pad (and lower leg [20]). Typically, energy input was varied and resulting maximal lower leg compression was measured. Qualitatively, our results were similar to the results of these previous studies for most subjects.

**Fig 7:**
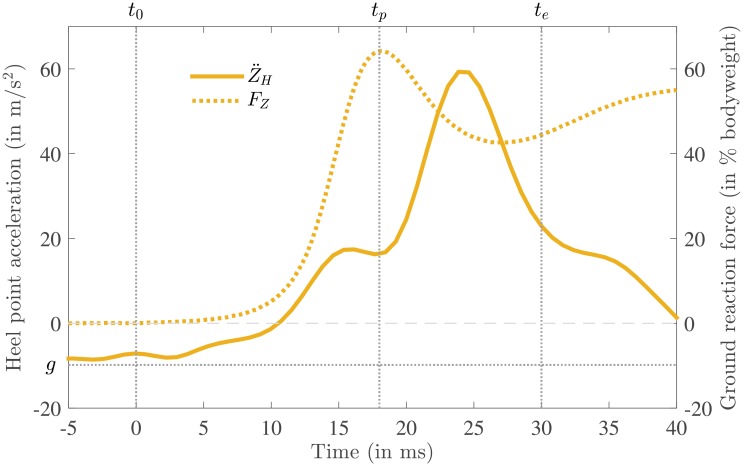
A typical example of the vertical heel point acceleration ${\ddot{Z}}_{H}$ in the same figure as vertical ground reaction force *F*_*Z*_ as reference (same measurement as in Figs 3 and 4). As in Fig 4 the start of impact is shown by a vertical dotted line indicated by *t*_0_, the instant of the vertical force peak is indicated by *t*_*p*_ and the end of heel strike, which is defined as zero vertical heel velocity, by *t*_*e*_. Here also the gravitational acceleration *g* is shown.

In Fig 7 the vertical heel acceleration ${\ddot{Z}}_{H}$ is shown together with the vertical GRF *F*_*Z*_ for the same measurement as shown in Fig 4. By comparing the behavior of vertical GRF *F*_*Z*_ to the behavior of the vertical heel point acceleration ${\ddot{Z}}_{H}$ it is clear that the peak in the acceleration occurs considerably later than the peak in the GRF.

## Discussion

In this study, we estimated the mechanical energy absorption during heel strike, by directly calculating the work done by the GRF on the foot by using the force-integral method. Our results on the relation between foot-ankle compression and compressive energy absorption are similar to previous mechanical lower leg property results [20] collected by [26]. Similar values for energy absorption results, due the vertical force only, were obtained from an improved application of the effective mass model of [15]; we found no significant difference in the results using the improved application of the effective foot mass model and the vertical component of the force-integral method. We did however find significant differences between these methods and the (total) force-integral method and the original effective foot mass model. We were able to reproduce very similar results using the original effective mass model to those reported by [15], however these are substantially lower than the values obtained using the other methods in this study.

Without synchronized kinematic and force data, [15] had to make assumptions about the relation between force data and kinematic data during heel strike. A key assumption in their approach was that the vertical heel velocity is zero at the instant of the impact peak and chose this instant as the final instant in their analysis. However this assumption is not consistent with our measurements and should be expected to be incorrect on theoretical grounds whenever the relative damping is non-negligible [31]. The observation that application of the original effective foot mass model in this study yielded values for energy absorption that are similar to those reported by [15] suggests that our heel point adequately represents the heel center studied by [15]; the observation that the original effective mass model yielded values for energy absorption that are substantially lower than those obtained with both the improved application of the effective mass model and the force-integral method proposed here, indicates that the erroneous assumption in the original effective mass model had substantial consequences for the estimated energy absorption.

A possible source of overestimation of the foot-ankle deformation is skin motion artifact, since the markers could both have moved downward relative to the bone after impact. However we saw no evidence of damped oscillation after impact in the raw marker data (raw data can be found at [19]) and the marker distance variation was negligible. It is therefore assumed that skin movement artifact is not the dominant error source. Another limitation of our research is that we assume that the contact area during heel strike is a constant point, whereas in reality rolling of the contact point, albeit with small amplitude, is likely to occur during heel strike. This means that a small part of the estimated foot-ankle deformation, should be in fact attributed to the movement of the contact point in the local coordinate system due to rolling of the contact point. Furthermore, analysis of the sensitivity of energy absorption for the position of the heel point has indicated that the methods for determining energy absorption proposed here do not depend strongly on the choice of the heel contact point. We therefore expect that these limitations will only have a relatively small contribution to the methodical error.

Our experimental approach depends on the assumption that the lower leg plus foot constitute a single body with constant configuration (no joint rotation) during heel strike; based on this assumption, the time-invariant location of the heel point in the local *xyz* coordinate system was determined. This assumption was previously made by [15], and was supported by inspection of high speed video data of the foot during heel strike, made during pilot experiments [19]. However we would reason that the error in our estimates is dominated by the neglected ankle rotation during initial contact, which is expected to be smaller than 5° [32]. Estimating the distance of the heel point to the ankle at 60 mm, this implies that in the worst case the heel point would move 5 mm in the local coordinate system due to the neglected ankle rotation. Considering that the vector from ankle joint axis to heel point is oriented nearly vertically during heel strike, we expect the effect of the ankle joint rotation on vertical displacement of the heel point to be very small. This would result in a small overestimation of the foot-ankle deformation and therefore also a small overestimation on the energy absorption estimation. We would therefore suggest to investigate other marker configurations, with at least one marker on the foot, in particular when studying abnormal gait patterns. We expect that the limitations discussed above result in a small bias in the results of both methods, therefore the comparison between methods is not compromised.

The compressive mechanical characteristics of the heel and lower leg (energy absorption as a function of maximum lower leg compression [20]) as estimated in this study are quite similar to those reported in previous research [26]. For some subjects the foot-ankle compression is slightly higher in the current study. This could be due to the fact that body mass and BMI of the participants in this study varied widely, whereas half of the data points reported in [26] were obtained from a single male recreational runner. Furthermore, activities such as running have been shown to influence the mechanical heel pad properties [33]. Differences could also be related to methodological issues, both in our study and in the studies evaluated by [26].

Given that the vertical component of the force-integral method proposed in this study and the improved application of the effective mass model yield similar values for energy absorption, one may ask which of these two methods is to be preferred in future studies. It is clear that the force-integral method entails less assumptions than the effective mass method, and on that ground alone the force-integral method is to be preferred. Furthermore, both methods require tightly synchronized high-frequency force and position data. The improved application of the effective mass model could possibly be useful in answering other research questions related to foot strike mechanics. However, we note that the movement of the heel point (which is assumed to represent the point mass in the effective mass model) during heel strike is not correctly predicted by the improved application of the effective mass model; in particular, the acceleration of the heel point is by no means proportional to the GRF, as they should be according to Newton’s second law. The force-integral method has as added strength that the total energy absorption due to heel strike, including the approximate shear losses corresponding to the lateral and anterior components of the GRF, can be estimated. All together, we suggest to use the force-integral method outlined in this study in future attempts to quantify the energy absorbed by the foot or heel pad during heel strike.

It should be noted that in this study we have only quantified the amount of energy absorbed by the heel pad, foot and ankle during its compression in the impact stage, the initial foot-ground contact. In later stages of the stance phase other parts of the heel and the rest of the foot will deform and absorb energy. This study sheds no light on the question to what extent this energy is buffered in elastic structures, allowing it to be returned to the body at a later stage, and to what extent it is converted into heat by viscous structures. However, this issue has been addressed by [26], who suggest that 75–95% of the energy absorbed by the heel pad and lower leg is dissipated; only a small fraction of the energy is stored in elastic structures. It is an open question if the elastic energy stored in the foot can be effectively returned to the system at a later stage of the stance phase. Although it has been shown that energy stored in a rotational spring at the ankle can be beneficial to the metabolic cost of walking [34, 35], it remains to be investigated to what extent this occurs for elastic energy stored in the heel pad, foot and ankle. At this point, we estimate the mechanical energy loss per step due to heel strike to be about 3.8 J.

Results of this study have implications for the importance of energy loss during heel strike in the energetics of human walking. According to this study, energy loss is around 3.8 J of mechanical energy per step during barefoot steady-motion level walking at preferred velocity. Assuming a mechanical efficiency for positive muscle fiber work of 20% [18], and considering that the total metabolic energy expenditure per step is in the order of 120 J [6], this corresponds to 15–20% of the total metabolic energy expended per step. In contrast to previous suggestions, mechanical energy lost during foot-ground contact contributes substantially to the metabolic energy cost of walking.

## Conclusion

We conclude that direct estimation of the work done by the ground reaction force is possible and preferable over the use of the effective foot mass model. The mechanical energy loss of heel strike is around 3.8 J for preferred walking speeds (≈ 1.3 ^m^/_s_), which contributes to about 15–20% of the overall metabolic cost of transport.

## References

[1] Passmore R, Durnin J. Human energy expenditure. Physiological Reviews. 1955;35(4):801–840. doi: 10.1152/physrev.1955.35.4.801 13266530 10.1152/physrev.1955.35.4.801

[2] Olney SJ, Monga TN, Costigan PA. Mechanical energy of walking of stroke patients. Archives of Physical Medicine and Rehabilitation. 1986;67(2):92–98. doi: 10.1016/0003-9993(86)90109-7 3954572 10.1016/0003-9993(86)90109-7

[3] Eng JJ, Chu KS, Dawson AS, Kim CM, Hepburn KE. Functional Walk Tests in Individuals With Stroke. Stroke. 2002;33(3). doi: 10.1161/hs0302.104195 11872900 10.1161/hs0302.104195

[4] Platts MM, Rafferty D, Paul L. Metabolic cost of overground gait in younger stroke patients and healthy controls. Medicine and Science in Sports and Exercise. 2006;38(6):1041–1046. doi: 10.1249/01.mss.0000222829.34111.9c 16775542 10.1249/01.mss.0000222829.34111.9c

[5] Zajac FE, Neptune RR, Kautz SA. Biomechanics and muscle coordination of human walking Part II: Lessons from dynamical simulations and clinical implications. Gait & Posture. 2003;17(1):1–17. doi: 10.1016/S0966-6362(02)00069-3 12535721 10.1016/s0966-6362(02)00069-3

[6] Kuo AD, Donelan JM, Ruina A. Energetic Consequences of Walking Like an Inverted Pendulum: Step-to-Step Transitions. Exercise and Sport Sciences Reviews. 2005;33(2):88–97. doi: 10.1097/00003677-200504000-00006 15821430 10.1097/00003677-200504000-00006

[7] Neptune RR, Kautz S, Zajac F. Contributions of the individual ankle plantar flexors to support, forward progression and swing initiation during walking. Journal of Biomechanics. 2001;34(11):1387–1398. doi: 10.1016/S0021-9290(01)00105-1 11672713 10.1016/s0021-9290(01)00105-1

[8] Neptune RR, Zajac FE, Kautz SA. Muscle mechanical work requirements during normal walking: the energetic cost of raising the body’s center-of-mass is significant. Journal of Biomechanics. 2004;37(6):817–825. doi: 10.1016/j.jbiomech.2003.11.001 15111069 10.1016/j.jbiomech.2003.11.001

[9] Kuo AD. The six determinants of gait and the inverted pendulum analogy: A dynamic walking perspective. Human Movement Science. 2007;26(4):617–656. doi: 10.1016/j.humov.2007.04.003 17617481 10.1016/j.humov.2007.04.003

[10] Neptune RR, Sasaki K, Kautz SA. The effect of walking speed on muscle function and mechanical energetics. Gait & Posture. 2008;28(1):135–143. doi: 10.1016/j.gaitpost.2007.11.004 18158246 10.1016/j.gaitpost.2007.11.004 PMC2409271

[11] Neptune RR, Zajac FE, Kautz SA. Author’s response to comment on “Contributions of the individual ankle plantar flexors to support, forward progression and swing initiation during walking” (Neptune et al., 2001) and “Muscle mechanical work requirements during normal walking: The energetic cost of raising the body’s center-of-mass is significant” (Neptune et al., 2004). Journal of Biomechanics. 2009;42(11):1786–1789. doi: 10.1016/j.jbiomech.2009.04.029 20606765 10.1016/j.jbiomech.2009.04.029 PMC2896326

[12] Kuo AD, Donelan JM. Comment on “Contributions of the individual ankle plantar flexors to support, forward progression and swing initiation during walking” (Neptune et al., 2001) and “‘Muscle mechanical work requirements during normal walking: The energetic cost of raising the body’s center-of-mass is significant” (Neptune et al., 2004). Journal of Biomechanics. 2009;42(11):1783–1789. 19482286 10.1016/j.jbiomech.2009.03.054 PMC4146491

[13] Zelik KE, Takahashi KZ, Sawicki GS. Six degree-of-freedom analysis of hip, knee, ankle and foot provides updated understanding of biomechanical work during human walking. The Journal of experimental biology. 2015;218(6):876–886 doi: 10.1242/jeb.115451 25788726 10.1242/jeb.115451

[14] Riddick RC, Kuo AD. Soft tissues store and return mechanical energy in human running. Journal of Biomechanics. 2016;49(3):436–441 doi: 10.1016/j.jbiomech.2016.01.001 26806689 10.1016/j.jbiomech.2016.01.001 PMC6224324

[15] Chi KJ, Schmitt D. Mechanical energy and effective foot mass during impact loading of walking and running. Journal of Biomechanics. 2005;38(7):1387–1395. doi: 10.1016/j.jbiomech.2004.06.020 15922749 10.1016/j.jbiomech.2004.06.020

[16] Lieberman DE, Venkadesan M, Werbel WA, Daoud AI, D’Andrea S, Davis IS, et al Foot strike patterns and collision forces in habitually barefoot versus shod runners. Nature. 2010;463(7280):531–535. doi: 10.1038/nature08723 20111000 10.1038/nature08723

[17] Ker RF, Bennett MB, Alexander RM, Kester RC. Foot strike and the properties of the human heel pad. Proceedings of the Institution of Mechanical Engineers Part H, Journal of engineering in medicine. 1989;203(4):191–196. doi: 10.1243/PIME_PROC_1989_203_038_01 2701955 10.1243/PIME_PROC_1989_203_038_01

[18] Whipp BJ, Wasserman K. Efficiency of muscular work. J Appl Physiol. 1969;26(5):644–648. doi: 10.1152/jappl.1969.26.5.644 5781619 10.1152/jappl.1969.26.5.644

[19] Baines PM, Schwab AL, van Soest AJ. PMBaines/ExperimentalData_EnergyAbsorptionDuringHeelStrike: Experimental data and processing scripts; 2018. Available from: https://github.com/PMBaines/ExperimentalData_EnergyAbsorptionDuringHeelStrike.

[20] Aerts P, Ker R, De Clercq D The mechanical properties of the human heel pad: a paradox resolved Journal of Biomechanics. 1995;24(11):1299–1308. doi: 10.1016/0021-9290(95)00009-7 10.1016/0021-9290(95)00009-7 8522543

[21] Cappozzo A, Catani F, Leardini A, Benedetti MG, Della Croce U. Position and orientation in space of bones during movement: experimental artefacts. Clinical Biomechanics. 1996;11(2):90–100. doi: 10.1016/0268-0033(95)00046-1 11415604 10.1016/0268-0033(95)00046-1

[22] Winter DA. Biomechanics and motor control of human movement. New York: Wiley; 1990.

[23] Kwon YH. Center of Pressure; 1998. Available from: http://www.kwon3d.com/theory/grf/cop.html.

[24] Greenwood DT. Advanced dynamics. Cambridge University Press; 2006.

[25] Bohannon RW, Williams Andrews A. Normal walking speed: a descriptive meta-analysis. Physiotherapy. 2011;97(3):182–189. doi: 10.1016/j.physio.2010.12.004 21820535 10.1016/j.physio.2010.12.004

[26] De Clercq D, Aerts P, Kunnen M. The mechanical characteristics of the human heel pad during foot strike in running: An in vivo cineradiographic study. Journal of Biomechanics. 1994;27(10):1213–1222. doi: 10.1016/0021-9290(94)90275-5 7962009 10.1016/0021-9290(94)90275-5

[27] Kinoshita H, Ogawa T, Arimoto K, Kuzuhara K, Ikuta K. Shock absorbing characteristics of human heel properties. Journal of Biomechanics. 1992;25(7):806 doi: 10.1016/0021-9290(92)90565-I

[28] Valiant GA. A determination of the mechanical characteristics of the human heel pad in vivo. The Pennsylvania State University, University Park, PA; 1984.

[29] Cavanagh P, Valiant G, Misevich K. Biological aspects of modeling shoe/foot interaction during running. Sport shoes and playing surfaces. 1984; p. 24–46.

[30] Aerts P, De Clercq D. Deformation characteristics of the heel region of the shod foot during a simulated heel strike: the effect of varying midsole hardness. J Sports Sci. 1993;11(5):449–61. doi: 10.1080/02640419308730011 8301705 10.1080/02640419308730011

[31] Rao SS. Mechanical vibrations. Prentice Hall; 2010.

[32] Kadaba MP, Ramakrishnan HK, Wootten ME. Measurement of Lower-Extremity Kinematics during Level Walking. Journal of Orthopaedic Research. 1990;8(3):383–392. doi: 10.1002/jor.1100080310 2324857 10.1002/jor.1100080310

[33] Challis JH, Murdoch C, Winter SL. Mechanical properties of the human heel pad: A comparison between populations Journal of Applied Biomechanics. 2008;24(4):377–381. doi: 10.1123/jab.24.4.377 19075307 10.1123/jab.24.4.377

[34] Alexander RM, Bennet-Clark H. Storage of elastic strain energy in muscle and other tissues. Nature. 1977;265(5590):114–117. doi: 10.1038/265114a0 834252 10.1038/265114a0

[35] Collins SH, Wiggin MB, Sawicki GS. Reducing the energy cost of human walking using an unpowered exoskeleton. Nature. 2015;522:212–215. doi: 10.1038/nature14288 25830889 10.1038/nature14288 PMC4481882

